# Burial of microplastics in freshwater sediments facilitated by iron-organo flocs

**DOI:** 10.1038/s41598-021-02748-4

**Published:** 2021-12-15

**Authors:** Rico Leiser, Maja Schumann, Tallent Dadi, Katrin Wendt-Potthoff

**Affiliations:** Department of Lake Research, Helmholtz Centre for Environmental Research, Brückstraße 3a, 39114 Magdeburg, Germany

**Keywords:** Environmental sciences, Limnology, Biogeochemistry

## Abstract

Microplastics are ubiquitous in standing freshwater bodies, consequently lakes and reservoirs may be important sinks for these contaminants. However, the mechanisms governing the deposition of microplastics and their interactions with the sediments are understudied. We demonstrate how aggregation-based transport facilitates the sinking and infiltration of buoyant microplastics into freshwater reservoir sediments by employing experiments with intact sediment cores. Buoyant polyethylene microplastics were rapidly (1–4 h) incorporated into sinking iron-organic aggregates, followed by swift deposition into sediments. Ingression of microplastic bearing flocs into sediments was completed within 6 days and led to stable deposition of the incorporated particles for at least 2 months. Most microplastics were deposited in the top 2 cm of the sediments and few particles (5–15%) were re-released into the water. Our results show at least 85% burial of microplastics, indicating the significant role of freshwaters with low flow velocities in reducing microplastic loads to the oceans.

## Introduction

Microplastics are particulate anthropogenic pollutants (< 5 mm) frequently found in the sediments of many lakes^1^ and reservoirs^2^ worldwide. Most of these plastics originate from rivers, flowing into the standing water bodies. The reduction of flow velocity leads to the settling of microplastics^3^, alongside with other particulate matter^4^ into the bottom sediments^5^. However, this only holds true for microplastic particles with high densities (ρ > 1.0 g cm^−3^) which allows their sedimentation in water^6^. Still, initially floating, low density (ρ < 1.0 g cm^−3^) polymer types such as polyethylene (PE) are among the most common microplastics retrieved from freshwater sediments^7^. This implies the existence of processes governing the sedimentation of buoyant microplastics in freshwater lakes and reservoirs. Such processes are aggregation^8^, biofouling^9^ and mineral formation^10^ affecting both high and low density microplastics. The microplastic particles may aggregate with microalgae cells^11^, cyanobacteria^12^, diatoms^13^ or transparent exopolymeric particles^8^ (TEP) leading to the sinking of initially buoyant polymers.

Large sinking aggregates (> 1 mm) composed of organic debris and inorganic particles^14^ are commonly found in lakes (“lake snow”^15^). Depending on the prevailing biogeochemical conditions “lake snow” may contain high proportions of iron oxy(hydroxides)^16,17^. The organic matter in such flocs consists of microbes^18^ and of co-precipitated dissolved or particulate organic carbon from the water^19^. Especially during lake stratification^20^ or autumnal lake mixing^21^ such iron-organo aggregates can be present in the water column. Iron-rich aggregates are usually formed at the upper part of the oxycline where anoxic water rich in ferrous iron comes into contact with oxic water. This leads to the oxidation of the ferrous to ferric iron^22^ which subsequently precipitates in the form of positively charged amorphous iron oxy(hydroxides) colloids^23,24^. These colloids form large and sinking flocs by aggregating with the net-negatively charged organic material present in lake water^16,24^. Such flocs may also be formed by the rapid breakdown of lake summer stratification^25^. This results in the complete mixing of the anoxic hypolimnion with the oxic epilimnion. The resulting iron-organo flocs are dispersed through the whole water column and co-precipitate microbial cells^21^, nutrients^26^ or heavy metals^27^. It was previously reported that lake mixing leads to aggregation of buoyant planktonic *Microcystis* colonies with iron flocs, transporting them to the sediment^21^. These mixing events might even aggregate and sink buoyant, negatively charged PE microplastics through aggregating with positively charged iron containing colloids^10,24^. Iron flocs formed in lakes are susceptible to microbial iron reduction^28^ especially once they reach the sediment^29^. Reduction might lead to the dissolution of the ballasting iron oxy(hydroxides), followed by floc disintegration^21^ and, if microplastics are enclosed, to microplastic liberation. Hence post-deposition floc stability might be crucial for the permanent removal of microplastics from the water-column. Iron reduction is more intense during summer anoxia compared to times in which the hypolimnion is oxygenated^30^. Therefore iron floc stability and microplastic release might depend on the prevailing redox condition in the hypolimnion.

In this study, we explored how the aggregation of PE microplastics into sinking iron-organo flocs affects their long-term deposition in freshwater reservoir sediments, which is a crucial step for the complete understanding of this globally important microplastic sink. We hypothesized that iron-organo floc formation can be simulated in the laboratory and that the incorporation of PE microplastics into such aggregates leads to sinking of this buoyant polymer. In addition, we assumed that the size and shape of the particles govern their enclosure rate into iron flocs. This was tested by amending surface water from a eutrophic reservoir with the iron flocculent Fe(II)SO_4_ and PE microplastics of three different shapes (fragments of four different size classes, fibers and spheres). Furthermore we tried to elucidate the fate of such microplastic bearing iron-organo flocs once reaching the bottom sediments of the water body. The first few mm of sediments and the overlying water column of lakes and reservoirs might be anoxic or oxic, depending on the season. With iron being a redox-sensitive element, we hypothesized that iron-organo-flocs containing PE microplastics lying on top of sediments will be stable under oxic conditions, while being disintegrated under anoxic conditions leading to microplastic release. This was tested with sediment cores from the eutrophic Bautzen reservoir (see Materials and methods section), which allow lab simulation of natural sediment processes due to their intact sediment surfaces. By addition of microplastic bearing iron-organo flocs to these sediment cores, we aimed to reconstruct the route of microplastics initially floating in the water column, into the sediments via an aggregation based transport mechanism.

## Results

### Iron-organo flocs formed by FeSO_4_ oxidation and their characteristics

**Figure 1:**
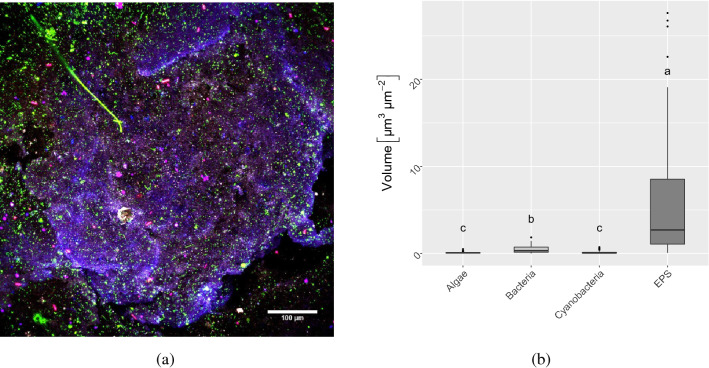
Floc analysis via CLSM, with (**a**) a representative iron-organo floc image showing bacteria (green), EPS (purple), cyanobacteria (pink) and algae (blue) and (**b**) the biovolumes of different microbial groups and EPS within 300 μM Fe flocs. Biovolumes were semi-quantitatively calculated from 50 individual images. Statistically significant differences (non-parametric bootstrapping differences of medians, 95% confidence interval (CI)) are displayed by small letters.

The addition of 100 or 300 µM FeSO_4_ to filtered Bautzen reservoir water (Supplementary Table 1) led to the formation of large and sinking iron-containing flocs within < 1 h (300 µM Fe) to 3–4 h (100 µM Fe) (Supplementary Fig. 1). The flocs formed by 100 µM Fe were generally fewer, smaller and had a lower density (Table 1) than the 300 µM Fe flocs. They were of reddish color emphasizing the high content of Fe (oxy)hydroxides (Supplementary Fig. 1). The main component was water (> 90%), while dry mass consisted of similar ratios of organic to inorganic components. The 300 µM Fe flocs had a significantly lower content of organics compared to the 100 µM flocs (ANOVA, F value: 52.56, *p* < 0.05). The inorganic content of the flocs was dominated by Fe, the organics consisted primarily of extracellular polymeric substances (EPS) enclosing microbial cells and minerals (Fig. 1). The sticky EPS can be considered as a binding agent gluing the cells and iron minerals together, thereby shaping the flocs’ gel-like appearance (Fig. 1a). Most cells within the flocs were identified as bacteria using Confocal Laser Scanning Microscopy (CLSM), but also small numbers of eukaryotic algae and cyanobacteria were present (Fig. 1b).

**Table 1:** Properties of flocs formed after addition of 100 µM and 300 µM Fe.

Parameter	100 µM Fe(II)	300 µM Fe(II)
Water content (%)	94.41 ± 0.9	96.1 ± 0.8
Dry mass (%)	5.6 ± 0.9	3.9 ± 0.8
Inorganics (%)	3.0 ± 0.5	2.6 ± 0.6
Organics (%)	2.6 ± 0.5	1.3 ± 0.2
Fe (%)	2.1 ± 0.7	2.9 ± 0.4
ESD (µm)	502 ± 132	3919 ± 700
Density (g cm^−3^)	1.005 ± 0.0006	1.015 ± 0.0016

Inorganic, organic and Fe contents refer to the wet mass of the flocs.

Displayed are the means and standard deviation of 6 replicates, except for equivalent spherical diameter^31^ (ESD) which was calculated from 30 individual flocs.

### Aggregation of different microplastic shapes into iron-organo flocs

**Figure 2:**
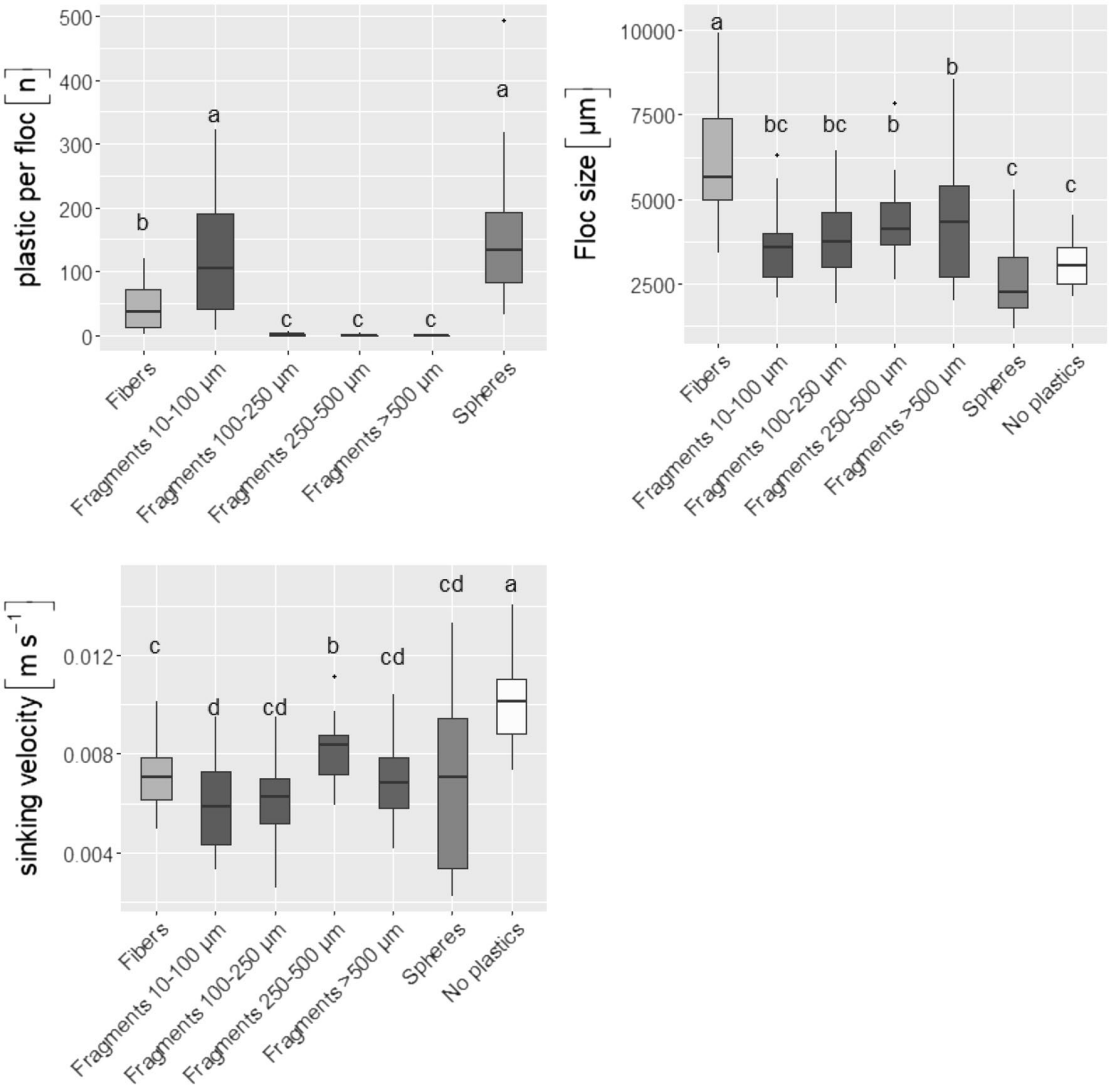
Plastic content, size and sinking velocity of flocs formed by addition of 300 μM Fe to Bautzen water containing different types of microplastics. Thirty individual flocs of each microplastic type were characterized. Letters indicate significant differences tested by using ANOVA (floc size, F value: 24.31, *p* < 0.05) or non-parametric bootstrapping (plastic content and sinking velocity, 95% CI).

Flocs formed by the addition of 300 µM Fe aggregated PE microplastics irrespective of their shape (Fig. 2a). Spheres and small fragments (10–100 µm) were incorporated more readily than fibers (Supplementary Table 2 & Fig. 2a). Only very few of the mid-sized fragments (100–250 µm) and almost none of the two largest fragment fractions (250–500 µm and > 500 µm) were taken up by the iron flocs (Supplementary Table 2 & Fig. 2a).

The floc sizes showed high variability with means ranging from 2632 ± 1666 µm (median 2263 µm, n: 30) for spheres to 6270 ± 1666 µm (median 5650 µm, n: 30) for fibers (Fig. 2b). Flocs formed with fibers had a pronounced elongated shape and contained macroscopic structures of entangled fibers (Supplementary Fig. 2). Beside this there were no clear differences in size between flocs with or without microplastics (Fig. 2b). By contrast, the presence of microplastics significantly reduced the sinking velocity of the flocs (non-parametric bootstrapping, 95% (CI), Fig. 2c). The mean sinking velocities of the flocs ranged from 0.006 ± 0.0018 m s^−1^ (median 0.0059 m s^−1^, n: 30) for fragments 10–100 µm to 0.01 ± 0.0017 m s^−1^ (median 0.0101 m s^−1^, n: 30) for no microplastics. However, given the high variability of the data, the absolute difference between the sinking velocities can be considered as minor, although being statistically significant.

Flocs formed by 100 µM Fe also aggregated PE spheres but showed lower precipitation of microplastics (28% of added spheres, mean, n: 3) compared to the 300 µM Fe flocs (99%, mean, n: 3). The lower aggregation efficiency was linked to the lower amount of flocs which were precipitated by this treatment (Supplementary Table 2). Normalized to the total floc mass the aggregation efficiency was similar for the 100 µM Fe compared to the 300 µM Fe treatment (7.4 vs 7.3 spheres per mg floc, mean, n: 3).

### Floc mediated microplastic transport into sediments

The sediment appeared soft and unconsolidated with grain sizes referring to clay (~ 3%), sand (~ 11%) and silt (~ 86%, Supplementary Table 3). The sediment had a homogeneous appearance showing no visible layering. The water content decreased from 92.97 ± 0.36% (mean ± SD, n: 10) in the top 2 cm to 86.431 ± 0.52% (mean ± SD, n: 10), while density increased from 1.079 ± 0.018 g cm^−3^ (mean ± SD, n: 10) to 1.132 ± 0.024 g cm^−3^ (mean ± SD, n: 10) from the top 2 cm to the bottom layer (8–11 cm; Supplementary Table 4).

To distinguish microplastics released or retained from small flocs generated at 100 µM Fe from that of large flocs formed at 300 µM Fe, initially buoyant spheres with different fluorescence labels were used (yellow: small flocs, red: large flocs). Experiments were started by adding iron flocs with PE to the overlying water of the sediment cores. Approximately 11,775 red spheres inside of 300 µM Fe flocs and 3231 yellow spheres inside of 100 µM Fe flocs were added to each core (Supplementary Table 2). The flocs settled through the water column and accumulated shortly at the sediment surface (Supplementary Fig. 3). Then they continued sinking through the sediment surface. Flocs were completely buried in the sediments and no longer visible at the surface after 24 h in the anoxic and 6 days in the oxic treatments (Supplementary Fig. 3). The anoxic cores showed extensive gas formation, which resulted in bubble release from the sediment. No obvious gas formation took place in the oxic cores, but bioturbation by burrowing chironomid larvae down to a depth of ~ 24 cm was observed.

**Figure 3:**
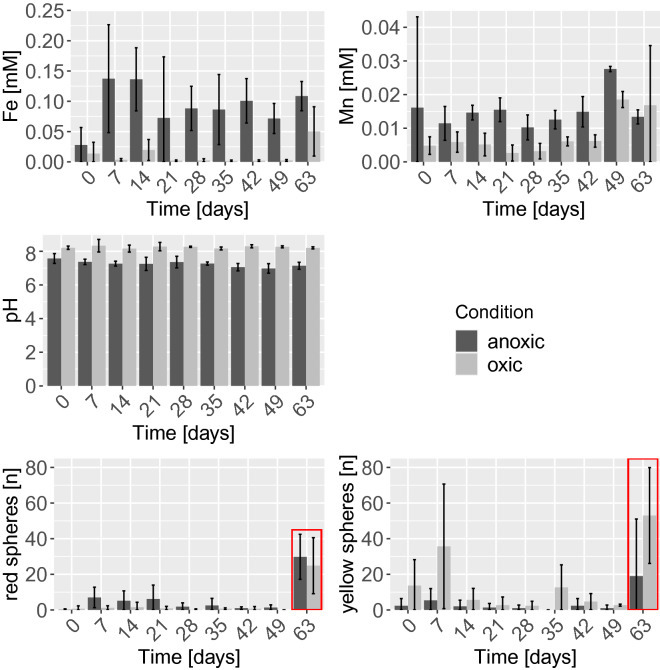
Results of weekly water phase samplings. Means and standard deviations are reported with 5 (anoxic) or 6 (oxic) replicates (July and October joined) for Fe and Mn concentrations, pH and release of red spheres from large 300 μM Fe flocs. Three replicates are reported for release of yellow spheres from small 100 μM Fe flocs (October). The red framing indicates the last sampling date, where sphere numbers were obtained by sampling the whole water phase above the sediment.

**Figure 4:**
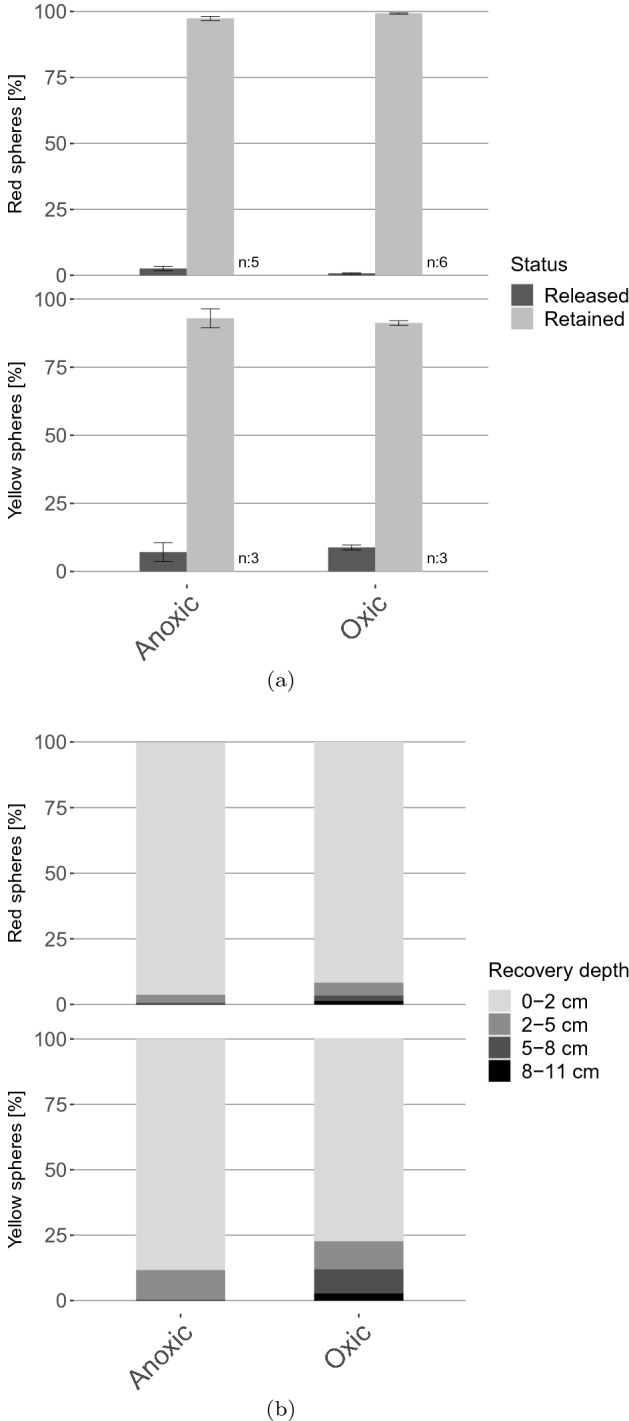
Recovery of PE spheres from sediment core experiments, with (**a**) ratio of released and retained spheres within the cores (July October) and (**b**) relative depth distribution of spheres (October). Results from October and July are combined for the large flocs (red spheres) whereas only results from October are presented for the small flocs (yellow spheres).

Iron reduction took place in the anoxic cores, while it was less pronounced in the oxic cores (Fig. 3). Microplastic release from the sediment was low throughout the whole experiment and not correlated to the iron release (Spearman’s rank correlation, red spheres rho: 0.45, yellow spheres rho: -0.28; Fig. 3). At the end of the experiment, 85% of the recovered yellow spheres and > 95% of the red spheres were found within the sediment (Fig. 4a and Supplementary Table 5 for absolute values). Hence the majority of microplastics were retained within the sediments and not released into the water phase during the experiment (Fig. 4a). Significantly more spheres (yellow and red; non-parametric bootstrapping, 95% CI) were recovered from the oxic compared to anoxic cores, indicating a sampling bias (Supplementary Table 5).

Most spheres were found within the uppermost 2 cm of the sediments in October cores (Fig. 4b). However, spheres were also detected in deeper layers. In the anoxic cores low numbers of spheres were present in the 2–5 cm layer. Their abundance decreased sharply with depth with only very few spheres recovered from 5 to 8 cm depth and no spheres recovered from the 8–11 cm layer of anoxic cores. In the oxic cores, spheres were found even in the deepest layer of 8 -11 cm depth, indicating a deeper burial compared to the anoxic cores. Comparatively high numbers of spheres were recovered from the 2–5 cm and 5–8 cm layer of the oxic cores. Similar patterns were also observed for July cores (Supplementary Fig. 4). Chironomid burrows were found throughout all layers of the oxic cores, averaging on 2–5 visible burrows per layer (Supplementary Fig. 5).

## Discussion

In this study we have presented evidence that PE microplastics are aggregated into sinking flocs formed by iron precipitates and organic material, irrespective of their shape. These flocs rapidly transported initially buoyant PE micro-spheres deep into freshwater sediments, leading to stable deposition given the long incubation time of 63 days.

The addition of ferrous sulfate to surface water of Bautzen Reservoir induced the formation of sinking flocs. This iron flocculation is a well-known process described in nature^23^ or employed in the context of water treatment technologies^32^. Flocs formed in our study were comparatively large, ranging from approximately 500 to > 3000 µm, whereas iron-organo lake aggregates or riverine composite suspended sediment particles^33^ are typically smaller with sizes from 60 µm^16^ to 457 µm^34^. However, organic and iron contents were comparable to natural flocs^16,35^. Iron-floc formation is usually considered a complex process involving the interaction of different microbial consortia forming complex 3-dimensional structures of EPS^36^ which enclose biogenic iron mineral grains and bacterial cells^28^. However, floc formation might also occur rapidly during seasonal lake mixing events without the involvement of complex microbial consortia^21^. This mode of formation might be more similar to the water treatment iron coagulation processes^24^ and to the experimental procedure we used. Even though we attempted to mimic natural conditions by using reservoir water, natural pH and partly natural iron concentrations^28^, the formed flocs will differ from natural ones. This should be considered when discussing the potential impact of iron flocculation in lakes on the fate of microplastics.

In addition, we could show that the iron-organo flocs readily incorporated buoyant microplastics. The incorporation depended on the size of the plastic particles. This is in line with other studies showing an increasing aggregation potential with decreasing microplastic particle size^37^, which might be explained by the higher collision frequency of smaller particles^38^. The overall aggregation efficiency of 300 µM Fe was higher for spheres compared to fragments or fibers, which might be explained by differences in size or polymer density. In another study iron concentrations of 370 µM removed up to 90% of small polystyrene microplastics from wastewater-effluents^39^, emphasizing the high potential of iron-organo flocs to trap microplastic.

The iron-organo flocs investigated in our study can be considered as a relevant type of aggregate capable of incorporating and sinking buoyant microplastics in freshwater. Previous studies showed that iron flocs formed during lake mixing could precipitate buoyant cyanobacteria colonies (size: 63 µm)^21^ and large PE microplastics (size: 4 × 4 × 0.15 mm)^10^. Although not proven in field experiments, it is likely that comparable aggregates forming in freshwater systems such as stratified lakes, reservoirs or flow-calmed zones of rivers are able to precipitate buoyant PE microplastics^10^ and thereby transport microplastics to the sediments or channel beds^40^. This is in line with many studies showing the removal of microplastics from the water column via aggregation mechanisms^41^. Iron-organic flocs are similar to other microplastic bearing aggregates such as marine^42^ or lake snow^15^, phytoplankton-^43^, EPS-^44^, and TEP-^8^ based flocs or riverine composite suspended sediment particles^33,34^ regarding their sizes, sinking velocities and densities.

Using sediment core experiments we could show that iron-organic aggregates containing initially buoyant microplastics rapidly subside into sediments and are not re-mobilized within 63 days. Hence we conclude that aggregation followed by sedimentation onto muddy sediments might lead to relatively stable deposition of microplastics in stagnant water bodies. Given that comparable flocs can also be found in fluvial environments, this deposition process is likely to be relevant for flow-calmed zones and floodplains of rivers or even estuaries.

Contrary to our initial hypothesis, anoxic conditions and iron reduction did not lead to a significant re-mobilization of microplastics from the sediments. Although more microplastics were released from anoxic compared to oxic cores, the majority of PE spheres remained in the sediments. This might be explained by the rapid downward transport of the iron flocs from the sediment surface into the sediments, by which microplastics were most likely trapped within the sediment matrix. Hence unlike iron oxide bound solutes such as pollutants or nutrients^25^, microplastics are not re-mobilized by anoxic conditions.

Sediments from the deepest part (~ 11 m) of Bautzen reservoir were used for our study. They were fine-grained, organic rich and rather unconsolidated. These are common properties of muddy sediments found in low current zones such as profundal zones of lakes^45^ and reservoirs^46^, oceanic basins (Baltic Sea^47^) or river estuaries. Recent studies showed that microplastics are often deposited in such low flow zones, making muddy sediments a likely depository of these anthropogenic particles^48^. In comparable deep-sea sediments from the Rockall Trough, microplastics were found in undisturbed layers with an approximate age of > 150 years^49^. This can only be explained by re-distribution of microplastics in the sediments after their deposition. Our results showed that aggregates containing microplastics can indeed easily penetrate the first cm of sediments. This offers a possible explanation for the unexpected appearance of microplastics in sediment layers deposited before the industrial production of the detected polymer types, which cannot be explained by bioturbation alone. The penetration of large particles through muddy sediments has been attributed to their gravitational force^50^ overcoming the cohesive force of the sediment particles^51^. The cohesiveness and density of sediments increase with depth, which will stop the downward movement of the flocs. It has been reported that large flocs formed by aluminum flocculation will accumulate to a depth of 10 cm in muddy lake sediments^52^. However in our study most PE spheres, which can be considered as proxy for the flocs, accumulated within the first 2 cm of the sediment. This is in line with findings indicating that phytoplankton aggregates accumulate and degrade within the upper few cm of lake sediments^53^. Still, we showed that a minor fraction has been transported deeper into the sediment reaching a depth of at least 11 cm. Interestingly, the smaller 100 µM Fe flocs reached deeper layers than the larger 300 µM Fe flocs, which is contradictory to the settling model driven by gravitational force. This might be explained by the higher potential of small flocs to migrate through small channels or cavities^54^. In conclusion, our study is in line with previous reports of microplastics^49^ or metal-organo flocs’ subsidence into sediments^52,55^. We showed that the rapid transport of buoyant PE into freshwater sediments can be facilitated by low density iron-rich organic aggregates. As the fate of aggregates strongly depends on the properties of the underlying sediment, the wider significance of our results is limited. Still, our findings should be applicable to most reservoirs and lakes because the type of sediment we investigated is broadly typical of these environments. More studies are needed to determine the response of other sediment types.

There is some evidence that PE spheres were transported deeper into the sediments of oxic cores compared to anoxic cores. This might have been caused by the presence of chironomid larvae in the oxic cores building long burrows (Supplementary Fig. 5). It has been previously reported that bioturbation by invertebrates will transport microplastics in fine-grained muddy sediments down- rather than upwards^47,56^. Therefore the burrowing and biodiffusive^57^ activity of the chironomids might explain the deeper distribution of spheres in the oxic cores. Our experimental setup excluded bioturbation by larger animals such as macroinvertebrates and benthivorous fish which exert complex and diverse modes of re-mobilization^58^. Based on our results the possible effect of bioturbation on the microplastic distribution in Bautzen reservoir cannot be evaluated completely.

Different limitations of the sediment core experiments need to be mentioned. Firstly, lower numbers of spheres were recovered from the anoxic compared to the oxic cores. This is particularly striking for the yellow spheres bound to 100 µM Fe flocs. It cannot be ruled out that the spheres or flocs were transported even deeper than the 11 cm used as lowest boundary. Extensive gas bubbles formed within the anoxic cores, which produced large voids in the deeper layers of the sediment. PE spheres might have fallen through these voids which brought them deeper into the cores than the sampled 11 cm layer. Considering that the missing spheres were not liberated from the sediments, but rather incorporated deeper than expected, this does not affect most of our statements. However, the assumption that spheres were transported deeper into the sediments of the oxic cores compared to the anoxic might not be justified.

Flocculation processes might contribute to the retention of microplastics transported from land to sea, as indicated by the presented results. Once initially buoyant microplastic is incorporated into sinking aggregates and reaches the sediment, it will rapidly be deposited inside the sediment matrix. Given the similiarties of iron-organo flocs compared to other lacustrine floc types or riverine composite suspended sediment particles, this process might play a role for the transport of microplastics in various freshwater environments. The observed accumulation of initially buoyant microplastics in fine sediments of riverbeds might also be influenced by their incorporation into larger and sinking aggregates. Excluding other processes such as current driven sediment re-suspension or bioturbation by larger organisms, this deposition might be stable even for longer time periods. This may not be valid for riverine environments were flooding events will re-suspend and transport microplastic particles downstream^59^. In lakes, deposition of microplastics during summer stratification could lead to permanent deposition, as no bioturbation takes place under anoxia, while several mm of sediments are settling over the microplastics during this time. Comparable conditions have been described leading to excellent fossilization of organic tissue or carcasses in stratified lakes^4^, for which undisturbed deposition over geological time-scale is required. This indicates that permanent and undisturbed deposition of microplastics in freshwater sediments is possible under certain conditions. However, lakes and reservoirs are typically located upstream of large urban centres which act as main source for environmental microplastics. This might diminish the revervoirs’ potential to act as important sink for these particulate contaminants. Still, the findings might draw more attention to the role of aggregation processes in reducing the plastic loads of aquatic systems in general and of rivers in particular. Comparable mechanisms might be relevant in the estuaries of large rivers with their flocculation zones and muddy sediments. Furthermore, factors leading either to re-suspension or to permanent burial of aggregated microplastics deposited in sediments should be investigated in further studies to improve the understanding of microplastic fate in the environment.

## Material and methods

### Study site and sampling

Bautzen Reservoir is located in Germany (size: 5.3 km^2^, mean depth: 7.4 m^60^) and shows labile summer stratification (June–September), with an anoxic hypolimnion frequently disrupted by strong winds^61^. Sediment cores and surface water samples were taken at the deepest point (~ 11 m) of the reservoir. Sediment cores were retrieved on 30th of July and 19th of October 2020 using a gravity corer (UWITEC, Austria, and PVC liners of 60 × 9 cm). Profiles of water parameters (Supplementary Fig. 6) were recorded using a multiparameter probe (Sea & Sun Technologies, Germany).

### Microplastic specification and preparation

PE spheres (d: 118 ± 6 µm, ρ: 0.98 g cm^−3^) spiked with fluorescent red Rhodamine B (RHBPMS-0.98 106–125 µm) or yellow stain (UVBGPMS-0.98 106–125 µm) from Cospheric, USA. PE fragments (ρ: 0.92 g cm^−3^, Alfa Aesar 9002-88-4) were passed through a sieving cascade (Retsch, Germany), to obtain four defined size ranges of fragments: > 500 µm ( ESD^31^): 727 ± 118 µm, n: 30), 250–500 µm (ESD: 466 ± 90 µm, n: 30), 100–250 µm (ESD: 234 ± 55 µm, n: 30) and 10–100 µm (ESD: 86 ± 26 µm, n: 30). The fragments were stained with fluorescent iDyePolyPink following established methods^62^. PE fibers (4600 × 24 µm, ρ: 0.92 g cm^−3^) were provided by Baumhueter extrusion GmbH, Germany (PB Eurofiber F-2106). More information on the microplastic particles are provided in Supplementary Table 6 and Supplementary Fig. 7.

### Floc formation and investigation of microplastic aggregation potential

Bautzen reservoir surface water was stored at 20° C in the dark and used within 2 weeks after sampling. Prior to use, the water was filtered through 10 µm stainless steel sieves and the filtrate was adjusted to pH 9.5 by 1 M NaOH (1 M) to reflect the alkaline conditions of the surface water during summer stratification (Supplementary Fig. 6). Experiments were conducted in triplicates per PE shape by amending 500 mL filtrate with 300 µL or 100 µL (final concentration: 300 or 100 µM) of a FeSO_4_ × 7 H_2_O stock solution (500 mM, pH 1.8) in airtight 1-L bottles. Thereafter 20 mg L^−1^ of microplastic fibers (9.0 × 10^3^ particles L^−1^), spheres (2.4 × 10^4^ particles L^−1^) or fragments (either fragments 10–100 µm: 6.6 × 10^4^ particles L^−1^, fragments 100–250 µm: 3.2 × 10^3^ particles L^−1^, fragments 250–500 µm: 2.2 × 10^2^ particles L^−1^ or fragments > 500 µm: 1.1 × 10^2^ particles L^−1^) were added separately to the respective bottles. Additionally, triplicates without added microplastics were used as control. The bottles were incubated on tumbling roller incubators (3–4 revolutions per minute, 20° C, RM5, M. Zipperer GmbH, Germany ) in ambient daylight for 24 h. Sinking velocity, size and plastic content of 10 individual flocs out of each bottle (triplicates per plastic type, n: 30) formed by 300 µM Fe were recorded following established methods^12^. The total amount of precipitated microplastic was quantified by repeating the bottle incubations, but recovering all flocs formed within the respective bottles. The flocs were gently washed with tap water, centrifuged (3000 rpm), weighed and extracted with 10 mL 1 M HCL (1 h, room temperature) followed by vigorous shaking (1 min) on a vortex mixer. The resulting suspension was filtered onto stainless steel filters and examined for their total plastic content by light microscopy.

Sinking velocities of flocs formed by 100 µM Fe could not be assessed due to their small size. Therefore solely total plastic content and average floc size (n: 30) were recorded.

### Floc characterization

The properties of iron flocs produced by 100 µM and 300 µM Fe without additional microplastics were further characterized by different methods. Densities of six individual flocs were determined by titration with NaCl solution (20% m/v) until neutral buoyancy, followed by pycnometer measurement of the resulting solution at 20 °C^12^. Water content, dry mass (60 °C, 24 h), loss on ignition (550 °C, 24 h) and total mass of flocs (n: 6) were measured after centrifugation (3000 rpm, 20 min) in pre-weighted conical centrifuge tubes. Fe content of the flocs’ wet mass was determined by dissolving defined, centrifuged (3000 rpm, 20 min) fractions (n: 6) in hydroxylamine hydrochloride-HCl (0.5/1 M) followed by measurement via ferrozine assay^63^. Confocal laser scanning microscopy (CLSM) was used to examine 5 randomly chosen spots each on 10 individual flocs produced with 300 µM Fe. The biovolumes of algae, bacteria, cyanobacteria and EPS were calculated from the resulting CLSM imaging datasets (n: 50).

### Experimental set-up of sediment incubation experiment

Cores were grouped into oxic and anoxic treatment with 3 replicates each. The anoxic triplicates were bubbled with N_2_ until depletion of O_2_ as measured via an internal oxygen optode (Pyroscience, Germany). Afterwards the anoxic cores were closed with custom-made covers preventing intrusion of oxygen and allowing anoxic sampling^64^. The oxic triplicates were closed with the same covers and bubbled constantly by air. The O_2_ levels in the cores were permanently recorded and adjusted on daily basis by N_2_ or O_2_ bubbling if necessary. Flocs formed by 300 µM Fe and containing red-fluorescent Rhodamine B PE spheres were produced as already described. In addition, for the October experiment, flocs formed by 100 µM Fe aggregating yellow-fluorescent PE spheres were prepared. The flocs were gently washed with tap water (three times) to remove non-aggregated microplastic spheres. The October cores were first supplemented with flocs containing red spheres and afterwards with flocs containing yellow spheres. The overlying water was exchanged by bottom to top through-flow of approximately 2 L of Bautzen surface water to remove spheres released by physical breakage of the flocs. Cores were photographed in 24 h intervals in the first week after floc application and in weekly intervals thereafter.

### Sampling procedure of sediment incubation experiment

Water samples for Fe(II), Mn, pH and released microplastics were taken in weekly intervals using syringes. Fe(II) was measured using ferrozine assay^63^, while Mn was measured using formaldoxime^65^. Microplastics were sampled by removing 120 mL of the uppermost part of the water-column using a syringe. The water was filtered over stainless steel filters (10 µm) and retained spheres were counted under a light microscope. The experiments were run for 63 days at 16 °C in the dark, after which the remaining water column was removed and examined for their plastic content. Afterwards the cores were sliced into sections of 0–2 cm, 2–5 cm, 5–8 cm and 8–11 cm using a sediment core cutter (Uwitec, Austria). The sections were transferred into centrifugation tubes and extracted by sonication followed by density separation with NaCl (20%, ρ: 1.56 g cm^−3^). The resulting suspension was centrifuged (3000 rpm, 15 min) and frozen by immersion in dry ice. The top layer of the frozen solution was transferred into conical centrifuge tubes by flushing with water and then filtered onto stainless steel grids. Microplastic contents of the layers were counted under the light microscope. The recovery rate was assessed by the addition of 1.2 × 10^4^ fluorescent PE spheres to sediment sections (0–2 cm, 2–5 cm, 5–8 cm, 8–11 cm) of a control core, followed by the already described extraction method. 1.06 × 10^4^ ± 3100 particles (mean ± sd, n: 4) were recovered from the sediments leading to recovery rate of 89.51 ± 2.61% (mean ± sd) for this method (data not shown). One anoxic core of the July experiment was lost due to inappropriate handling, resulting in lower sample numbers for the anoxic treatments. The bulk density, porosity, water content, dry mass, organic content and grain size distributions of two control cores were determined by standard methods^60^. Furthermore the Fe(II)/Fe(III) and Mn contents of the sediments were determined after extraction with HCl (1 M) and hydroxylamine hydrochloride-HCl (0.5/1 M) using ferrozine or formaldoxime assay, respectively.

### CLSM imaging

Flocs were visualized using CLSM in combination with different fluorescent dyes^12^. In brief, flocs were mounted in microscope chamber slides (Thermo Fisher Scientific) and stained. *Aleuria aurantia* lectin (Vector Laboratories, USA) labeled with Alexa Fluor 633 (Thermo Fisher Scientific, USA) was used to visualize the extracellular polymeric substances (EPS) of the flocs^66,67^. Bacteria were detected via SybrGreen staining, while algae and cyanobacteria were identified by the autofluorescence of their chlorophyll *a* or phycobilins, respectively. Imaging was done by a TCS SP5X upright microscope equipped with white laser and water-immersible lens (25x/0.95), controlled by LAS AF version 2.4.1 (Leica, Germany). The filter configurations used for excitation and emission are listed in Supplementary Table 7. Imaris (Bitplane) was used to visualize the images, which were printed by Photoshop (Adobe). Biovolumes of algae, bacteria, cyanobacteria and EPS were semi-quantitatively calculated employing an adaption of ImageJ^68^.

### Statistical analysis

Statistical testing was only conducted for datasets with a minimum sample size of 5 individual replicates. Q-Q plots were used to check for data normality. Bartlett’s test was used to test variance homogeneity prior to one-sided ANOVA (Type II) which was used to compare group means. Residual plots were examined to verify the reliability of the ANOVA. Group means were assumed to be significantly different from each other for *p* < 0.05. Tukey’s post-hoc test was computed for pair-wise comparison. Samples not meeting assumptions of the ANOVA were tested by non-parametric bootstrapping^69^. The 95% confidence intervals (CI) of median differences of 10,000 bootstrapped samples were reported. Differences in median CI higher or lower than zero were defined as significantly different from each other by 95% chance. Spearman’s rank correlation was used to calculate correlation coefficients. Software R^70^ was used for all statistical analysis and data visualizations.

## Supplementary Information

Supplementary Information.

## References

[1] Turner S, Horton AA, Rose NL, Hall C (2019). A temporal sediment record of microplastics in an urban lake, London, UK. J. Paleolimnol..

[2] Di M, Wang J (2018). Microplastics in surface waters and sediments of the Three Gorges Reservoir, China. Sci. Total Environ..

[3] Watkins L, McGrattan S, Sullivan PJ, Walter MT (2019). The effect of dams on river transport of microplastic pollution. Sci. Total Environ..

[4] Franzen JL (1985). Exceptional preservation of Eocene vertebrates in the lake deposit of Grube Messel (West Germany). Philos. Trans. R. Soc. Lond. B Biol. Sci..

[5] Enders K, Käppler A, Biniasch O, Feldens P, Stollberg N, Lange X, Fischer D, Eichhorn KJ, Pollehne F, Oberbeckmann S, Labrenz M (2019). Tracing microplastics in aquatic environments based on sediment analogies. Sci. Rep..

[6] Chubarenko I, Bagaev A, Zobkov M, Esiukova E (2016). On some physical and dynamical properties of microplastic particles in marine environment. Mar. Pollut. Bull..

[7] Merga LB, Redondo-Hasselerharm PE, Van den Brink PJ, Koelmans AA (2020). Distribution of microplastic and small macroplastic particles across four fish species and sediment in an African lake. Sci. Total Environ..

[8] Michels J, Stippkugel A, Lenz M, Wirtz K, Engel A (2018). Rapid aggregation of biofilm-covered microplastics with marine biogenic particles. Proc. Biol. Sci..

[9] Kaiser D, Kowalski N, Waniek JJ (2017). Effects of biofouling on the sinking behavior of microplastics. Environ. Res. Lett..

[10] Leiser R, Wu G-M, Neu TR, Wendt-Potthoff K (2020). Biofouling, metal sorption and aggregation are related to sinking of microplastics in a stratified reservoir. Water Res..

[11] Lagarde F, Olivier O, Zanella M, Daniel P, Hiard S, Caruso A (2016). Microplastic interactions with freshwater microalgae: Hetero-aggregation and changes in plastic density appear strongly dependent on polymer type. Environ. Pollut..

[12] Leiser R, Jongsma R, Bakenhus I, Möckel R, Philipp B, Neu TR, Wendt-Potthoff K (2021). Interaction of cyanobacteria with calcium facilitates the sedimentation of microplastics in a eutrophic reservoir. Water Res..

[13] Long M, Paul-Pont I, Hégaret H, Moriceau B, Lambert C, Huvet A, Soudant P (2017). Interactions between polystyrene microplastics and marine phytoplankton lead to species-specific hetero-aggregation. Environ. Pollut..

[14] Grossart HP, Simon M, Logan BE (1997). Formation of macroscopic organic aggregates (lake snow) in a large lake: The significance of transparent exopolymer particles, phytoplankton, and zooplankton. Limnol. Oceanogr..

[15] Grossart HP, Simon M (1993). Limnetic macroscopic organic aggregates (lake snow): Occurrence, characteristics, and microbial dynamics in Lake Constance. Limnol. Oceanogr..

[16] Reiche M, Lu S, Ciobotǎ V, Neu TR, Nietzsche S, Rösch P, Popp J, Küsel K (2011). Pelagic boundary conditions affect the biological formation of iron-rich particles (iron snow) and their microbial communities. Limnol. Oceanogr..

[17] Elliott AVC, Warren LA (2014). Microbial engineering of floc Fe and trace element geochemistry in a circumneutral, remote lake. Environ. Sci. Technol..

[18] Plach JM, Elliott AVC, Droppo IG, Warren LA (2011). Physical and ecological controls on freshwater floc trace metal dynamics. Environ. Sci. Technol..

[19] Pizarro J, Belzile N, Filella M, Leppard GG, Negre JC, Perret D, Buffle J (1995). Coagulation sedimentation of submicron iron particles in a eutrophic lake. Water Res..

[20] Bravidor J, Kreling J, Lorke A, Koschorreck M (2015). Effect of fluctuating oxygen concentration on iron oxidation at the pelagic ferrocline of a meromictic lake. Environ. Chem..

[21] Oliver RL, Thomas R, Reynold CS, Walsby AE (1985). The sedimentation of buoyant microcystis colonies caused by precipitation with an iron-containing colloid. Proc. R. Soc. B.

[22] Cornell R, Schwertmann U (2003). The Iron Oxides: Structure, Properties, Reaction, Occurrences and Uses.

[23] Tipping E, Woof C, Cooke D (1981). Iron oxide from a seasonally anoxic lake. Geochim. Cosmochim. Acta.

[24] Ma B, Xue W, Hu C, Liu H, Qu J, Li L (2019). Characteristics of microplastic removal via coagulation and ultrafiltration during drinking water treatment. Chem. Eng. J..

[25] Mortimer CH (1942). The exchange of dissolved substances between mud and water in lakes. J. Ecol..

[26] Deppe T, Benndorf J (2002). Phosphorus reduction in a shallow hypereutrophic reservoir by in-lake dosage of ferrous iron. Water Res..

[27] Díez S, Noonan GO, MacFarlane JK, Gschwend PM (2007). Ferrous iron oxidation rates in the pycnocline of a permanently stratified lake. Chemosphere.

[28] Elliott AVC, Plach JM, Droppo IG, Warren LA (2014). Collaborative microbial Fe-redox cycling by pelagic floc bacteria across wide ranging oxygenated aquatic systems. Chem. Geol..

[29] Venkateswaran K, Moser DP, Dollhopf ME, Lies DP, Saffarini DA, MacGregor BJ, Ringelberg DB, White DC, Nishijima M, Sano H, Burghardt J, Stackebrandt E, Nealson KH (1999). Polyphasic taxonomy of the genus *Shewanella* and description of *Shewanella oneidensis* sp. nov. Int. J. Syst. Bacteriol..

[30] Wendt-Potthoff K, Kloß C, Schultze M, Koschorreck M (2014). Anaerobic metabolism of two hydro-morphological similar pre-dams under contrasting nutrient loading (Rappbode Reservoir System, Germany). Int. Rev. Hydrobiol..

[31] Kaiser D, Estelmann A, Kowalski N, Glockzin M, Waniek JJ (2019). Sinking velocity of sub-millimeter microplastic. Mar. Pollut. Bull..

[32] Teh CY, Budiman PM, Shak KPY, Wu TY (2016). Recent advancement of coagulation–flocculation and its application in wastewater treatment. Ind. Eng. Chem. Res..

[33] Woodward JC, Walling DE (2007). Composite suspended sediment particles in river systems: Their incidence, dynamics and physical characteristics. Hydrol. Process..

[34] Droppo IG, Leppard GG, Flannigan DT, Liss SN (1997). The freshwater floc: A functional relationship of water and organic and inorganic floc constituents affecting suspended sediment properties. Water Air Soil Pollut..

[35] Elliott AVC, Plach JM, Droppo IG, Warren LA (2012). Comparative floc-bed sediment trace element partitioning across variably contaminated aquatic ecosystems. Environ. Sci. Technol..

[36] Mori JF, Ueberschaar N, Lu S, Cooper RE, Pohnert G, Küsel K (2017). Sticking together: Inter-species aggregation of bacteria isolated from iron snow is controlled by chemical signaling. ISME J..

[37] Shams M, Alam I, Chowdhury I (2020). Aggregation and stability of nanoscale plastics in aquatic environment. Water Res..

[38] Quik JTK, van De Meent D, Koelmans AA (2014). Simplifying modeling of nanoparticle aggregation-sedimentation behavior in environmental systems: A theoretical analysis. Water Res..

[39] Rajala K, Grönfors O, Hesampour M, Mikola A (2020). Removal of microplastics from secondary wastewater treatment plant effluent by coagulation/flocculation with iron, aluminum and polyamine-based chemicals. Water Res..

[40] Woodward J, Li J, Rothwell J, Hurley R (2021). Acute riverine microplastic contamination due to avoidable releases of untreated wastewater. Nat. Sustain..

[41] Kvale K, Prowe AEF, Chien CT, Landolfi A, Oschlies A (2020). The global biological microplastic particle sink. Sci. Rep..

[42] Maggi F (2013). The settling velocity of mineral, biomineral, and biological particles and aggregates in water. J. Geophys. Res. Oceans.

[43] Long M, Paul-Pont I, Hégaret H, Moriceau B, Lambert C, Huvet A, Soudant P (2015). Interactions between microplastics and phytoplankton aggregates: Impact on their respective fates. Mar. Chem..

[44] Möhlenkamp P, Purser A, Thomsen L (2018). Plastic microbeads from cosmetic products: An experimental study of their hydrodynamic behaviour, vertical transport and resuspension in phytoplankton and sediment aggregates. Elem. Sci. Anthr..

[45] Håkanson L (1981). Determination of characteristic values for physical and chemical lake sediment parameters. Water Resour. Res..

[46] Abraham J, Allen PM, Dunbar JA, Dworkin SI (1999). Sediment type distribution in reservoirs: Sediment source versus morphometry. Environ. Geol..

[47] Näkki P, Setälä O, Lehtiniemi M (2019). Seafloor sediments as microplastic sinks in the northern Baltic Sea—Negligible upward transport of buried microplastics by bioturbation. Environ. Pollut..

[48] Zobkov M, Belkina N, Kovalevski V, Zobkova M, Efremova T, Galakhina N (2020). Microplastic abundance and accumulation behavior in Lake Onego sediments: A journey from the river mouth to pelagic waters of the large boreal lake. J. Environ. Chem. Eng..

[49] Courtene-Jones W, Quinn B, Ewins C, Gary SF, Narayanaswamy BE (2020). Microplastic accumulation in deep-sea sediments from the Rockall Trough. Mar. Pollut. Bull..

[50] Huettel A, Ziebis W, Forster S (1996). Flow-induced uptake of particulate matter in permeable sediments. Limnol. Oceanogr..

[51] Wheatcroft RA (1992). Experimental tests for particle size-dependent bioturbation in the deep ocean. Limnol. Oceanogr..

[52] Łopata M, Augustyniak R, Grochowska J, Parszuto K, Tandyrak R, Wiśniewski G (2020). Behavior of aluminum compounds in soft-water lakes subjected to experimental reclamation with polyaluminum chloride. Water. Air. Soil Pollut..

[53] Schulz S, Conrad R (1995). Effect of algal deposition on acetate and methane concentrations in the profundal sediment of a deep lake (Lake Constance). FEMS Microbiol. Ecol..

[54] Rusch A, Huettel M (2000). Advective particle transport into permeable sediments—Evidence from experiments in an intertidal sandflat. Limnol. Oceanogr..

[55] Lewandowski J, Schauser I, Hupfer M (2003). Long term effects of phosphorus precipitations with alum in hypereutrophic Lake Süsser See (Germany). Water Res..

[56] Näkki P, Setälä O, Lehtiniemi M (2017). Bioturbation transports secondary microplastics to deeper layers in soft marine sediments of the northern Baltic Sea. Mar. Pollut. Bull..

[57] Baranov V, Lewandowski J, Romeijn P, Singer G, Krause S (2016). Effects of bioirrigation of non-biting midges (Diptera: *Chironomidae*) on lake sediment respiration. Sci. Rep..

[58] Adámek Z, Maršálek B (2013). Bioturbation of sediments by benthic macroinvertebrates and fish and its implication for pond ecosystems: A review. Aquac. Int..

[59] Hurley R, Woodward J, Rothwell JJ (2018). Microplastic contamination of river beds significantly reduced by catchment-wide flooding. Nat. Geosci..

[60] Kasprzak P, Benndorf J, Gonsiorczyk T, Koschel R, Krienitz L, Mehner T, Hülsmann S, Schultz H, Wagner A (2007). Reduction of nutrient loading and biomanipulation as tools in water quality management: Long-term observations on Bautzen Reservoir and Feldberger Haussee (Germany). Lake Reserv. Manag..

[61] Kerimoglu O, Rinke K (2013). Stratification dynamics in a shallow reservoir under different hydro-meteorological scenarios and operational strategies. Water Resour. Res..

[62] Karakolis EG, Nguyen B, You JB, Rochman CM, Sinton D (2019). Fluorescent dyes for visualizing microplastic particles and fibers in laboratory-based studies. Environ. Sci. Technol. Lett..

[63] Stookey LL (1970). Ferrozine—A new spectrophotometric reagent for iron. Anal. Chem..

[64] Dadi T, Harir M, Hertkorn N, Koschorreck M, Schmitt-Kopplin P, Herzsprung P (2017). Redox conditions affect dissolved organic carbon quality in stratified freshwaters. Environ. Sci. Technol..

[65] Burlage R, Atlas R, Stahl D (1998). Techniques in Microbial Ecology.

[66] Neu TR, Swerhone GDW, Lawrence JR (2001). Assessment of lectin-binding analysis for in situ detection of glycoconjugates in biofilm systems. Microbiology.

[67] Neu TR, Kuhlicke U, Lawrence JR (2002). Assessment of fluorochromes for two-photon laser scanning microscopy of biofilms. Appl. Environ. Microbiol..

[68] Staudt C, Horn H, Hempel DC, Neu TR (2004). Volumetric measurements of bacterial cells and extracellular polymeric substance glycoconjugates in biofilms. Biotechnol. Bioeng..

[69] Efron B, Tibshirani R (1986). Bootstrap methods for standard errors, confidence intervals, and other measures of statistical accuracy. Stat. Sci..

[70] R Development Core Team. *R: A Language and Environment for Statistical **Computing *(R Foundation for Statistical Computing, 2021).

