# Supplementary material for: Burial of microplastics in freshwater sediments facilitated by iron-organo flocs
Source: Sci Rep. 2021 Dec 15;11:24072. doi: 10.1038/s41598-021-02748-4 (PMC8674222; doi:10.1038/s41598-021-02748-4)
Supplement: Supplementary file 1 — Supplementary Information. [file 41598_2021_2748_MOESM1_ESM.pdf]

Burial of microplastics in freshwater sediments facilitated by iron-organo flocs

by Rico Leiser*[,1], Maja Schumann[1], Tallent Dadi[1] and Katrin Wendt-Potthoff[1]

[1]*Department of Lake Research, Helmholtz Centre for Environmental Research, Brückstraße 3a, 39114 Magdeburg, Germany*

*Corresponding author

E-mail address: rico.leiser@ufz.de

Supplementary Table 1: Water parameters of Bautzen reservoir surface water before and after the addition of various concentrations of $FeSO_4$ x 9 H_2O.

Parameter	Bautzen reservoir water before Fe precipitation	after 100 µM Fe addition	after 300 µM Fe addition	Unit
pH	7.7	7.7	7.7	-
total inorganic carbon	16.7	16.6	12.0	mg L^{-1}
dissolved organic carbon	7.24	4.96	4.45	mg L^{-1}
$Mn_{diss.}$	<0,007	0.027	0.125	mg L^{-1}
Fe_{diss}	0.015	0.083	0.024	mg L^{-1}
total phosphorus	0.13	0.027	0.029	mg L^{-1}
total nitrogen bound	1.69	1.21	1.09	mg L^{-1}
NO_{3}-N	0.740	0.542	0.564	mg L^{-1}
NH_{4}-N	0.238	<0,010	0.013	mg L^{-1}
soluble reactive phosphorus	0.083	<0,003	<0,003	mg L^{-1}
Si	3.77	3.48	3.40	mg L^{-1}

a)

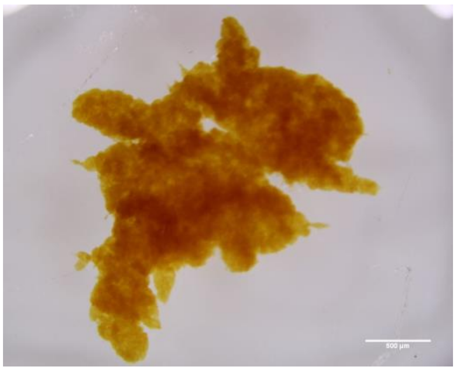

b)

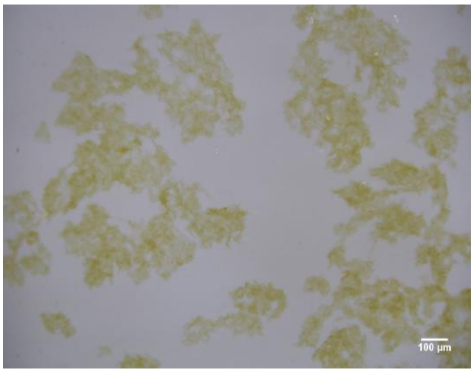

Supplementary Figure 1: Flocs formed after the addition of 300 µM (a) and 100 µM $FeSO_4$ (b) in water from Bautzen reservoir

Supplementary Table 2: Microplastic content of iron flocs formed after the addition of 300 µM or 100 µM $FeSO_4$. Displayed are the means ± standard deviations of 3 individual replicates.

Microplastics	Fe concentration in bottle [µM]	Plastic particles incorporated	Wet weight of precipitated flocs [mg]
Fibres	300	1948 ± 148 (37 %)	1057 ± 197
Spheres	300	11775 ± 1010 (99 %)	1604 ± 23
Fragments 10 – 100 µm	300	8810 ± 1763 (27 %)	727 ± 62
Fragments 100 - 250 µm	300	255 ± 142 (16 %)	1065 ± 155
Fragments 250 - 500 µm	300	8 ± 1 (4 %)	997 ± 104
Fragments > 500 µm	300	1 ± 1 (1 %)	978 ± 117
Spheres	100	3275 ± 1735 (28 %)	442 ± 88

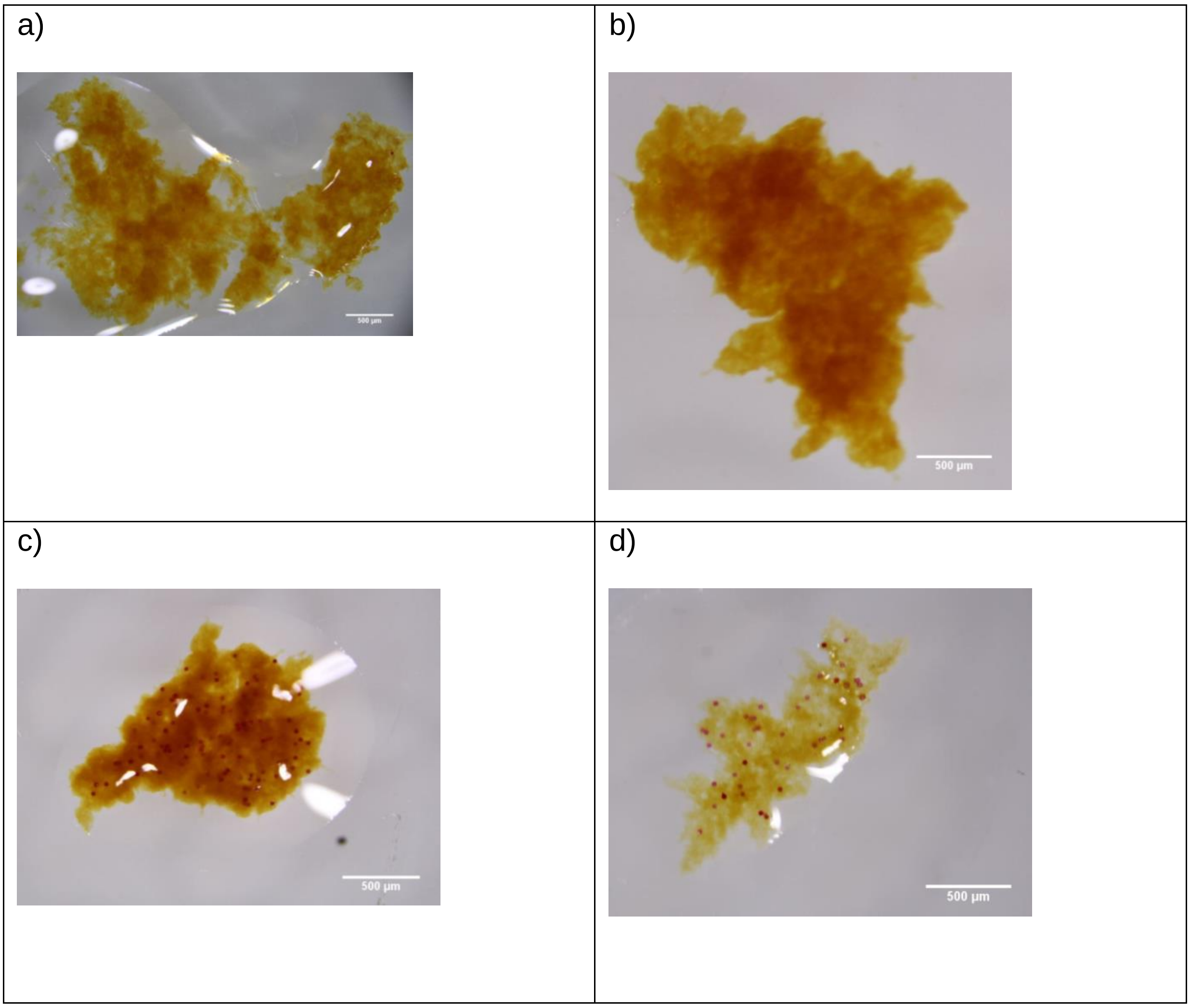

Supplementary Figure 2: Representative images of flocs formed by addition of different microplastic shapes and Fe concentrations, with a) showing 300 µM Fe and fibres, b) 300 µM Fe and small fragments (10-100 µm), c) 300 µM Fe and spheres and d) 100 µM Fe and spheres

Supplementary Table 3: Grain size distribution of two sediment cores from Bautzen reservoir. Sediments were classified according to [1] with grain sizes < 6 µm defined as clay, 6 – 63 µm defined as silt, and 63 - 2000 µm defined as sand.

Depth	Clay [%]	Silt [%]	Sand [%]
0 – 2 cm	2.72 ± 0.28	82.56 ± 2.67	13.96 ± 2.79
2 – 5 cm	2.40 ± 0.75	78.58 ± 1.24	19.02 ± 0.64
5 – 8 cm	3.53 ± 0.35	90.035 ± 0.20	6.43 ± 0.52
8 – 11 cm	3.54 ± 0.27	91.13 ± 0.58	5.33 ± 0.46

Supplementary Table 4: Physical properties of two sediment cores from Bautzen reservoir. The mean and standard deviation of 5 replicates per core (n: 10) are reported.

Depth	Density [kg m^{-3}]	Water content [%]	Dry mass [%]	Ash mass [%]
0 – 2 cm	1.079 ± 0.018	92.97 ± 0.36	7.03 ± 0.36	5.59 ± 0.31
2 – 5 cm	1.089 ± 0.013	92.42 ± 0.09	7.57 ± 0.09	5.91 ± 0.20
5 – 8 cm	1.108 ± 0.025	85.31 ± 0.08	14.69 ± 0.08	12.92 ± 0.31
8 – 11 cm	1.132 ± 0.024	86.431 ± 0.52	13.57 ± 0.52	12.25 ± 0.49

Core	After addition	After 24 h	After 6 days
Anoxic	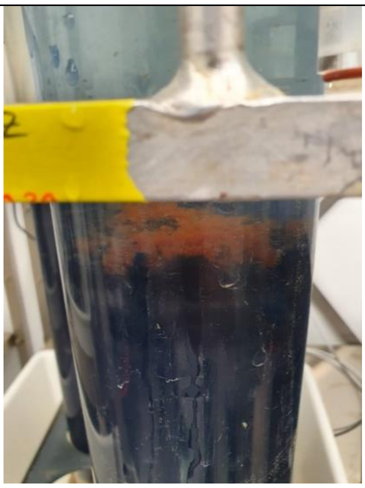	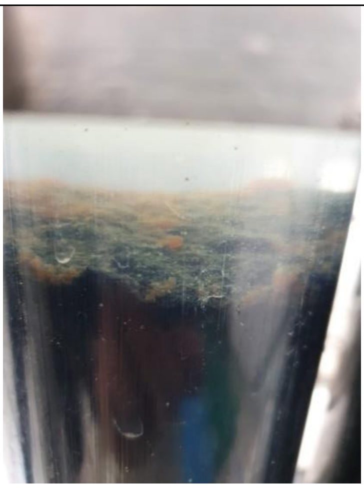	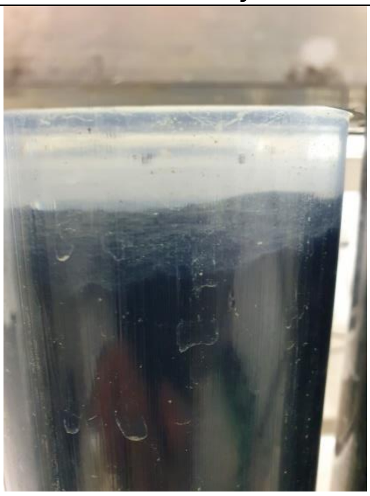
Oxic	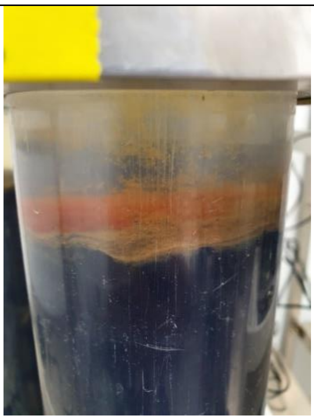	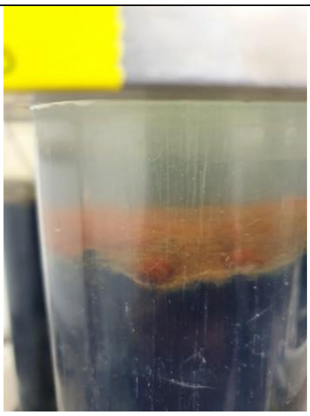	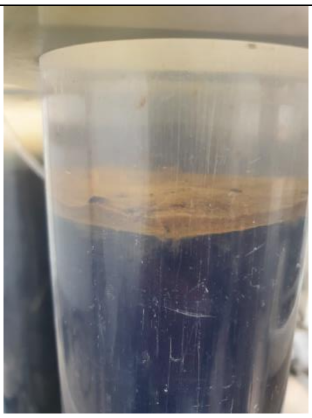

Supplementary Figure 3: Anoxic and oxic sediment cores from October experiment with added iron flocs, shortly after addition, 24 h after addition and 6 days after addition.

Supplementary Table 5: Recovery of microplastic spheres from sediment cores. N.a.: not available, this term is used for the cores from August, where no flocs formed by addition of 100 µM Fe labeled with yellow spheres were added. The status "released" refers to spheres found in the water phase, whereas "in sediment" refers to spheres extracted from the sediments after ending the experiment.

Core	Date	Status	red spheres released	red spheres in sediment	yellow spheres released	yellow spheres in sediment
1	August	anoxic	75	4862	n.a.	n.a.
2	August	anoxic	100	1303	n.a.	n.a.
3	October	anoxic	37	3835	2	18
4	October	anoxic	38	2670	99	869
5	October	anoxic	65	2949	2	7
6	August	oxic	52	5237	n.a.	n.a.
7	August	oxic	25	4477	n.a.	n.a.
8	August	oxic	68	3477	n.a.	n.a.
9	October	oxic	22	9496	146	2522
10	October	oxic	23	6677	166	2256
11	October	oxic	21	3897	87	146

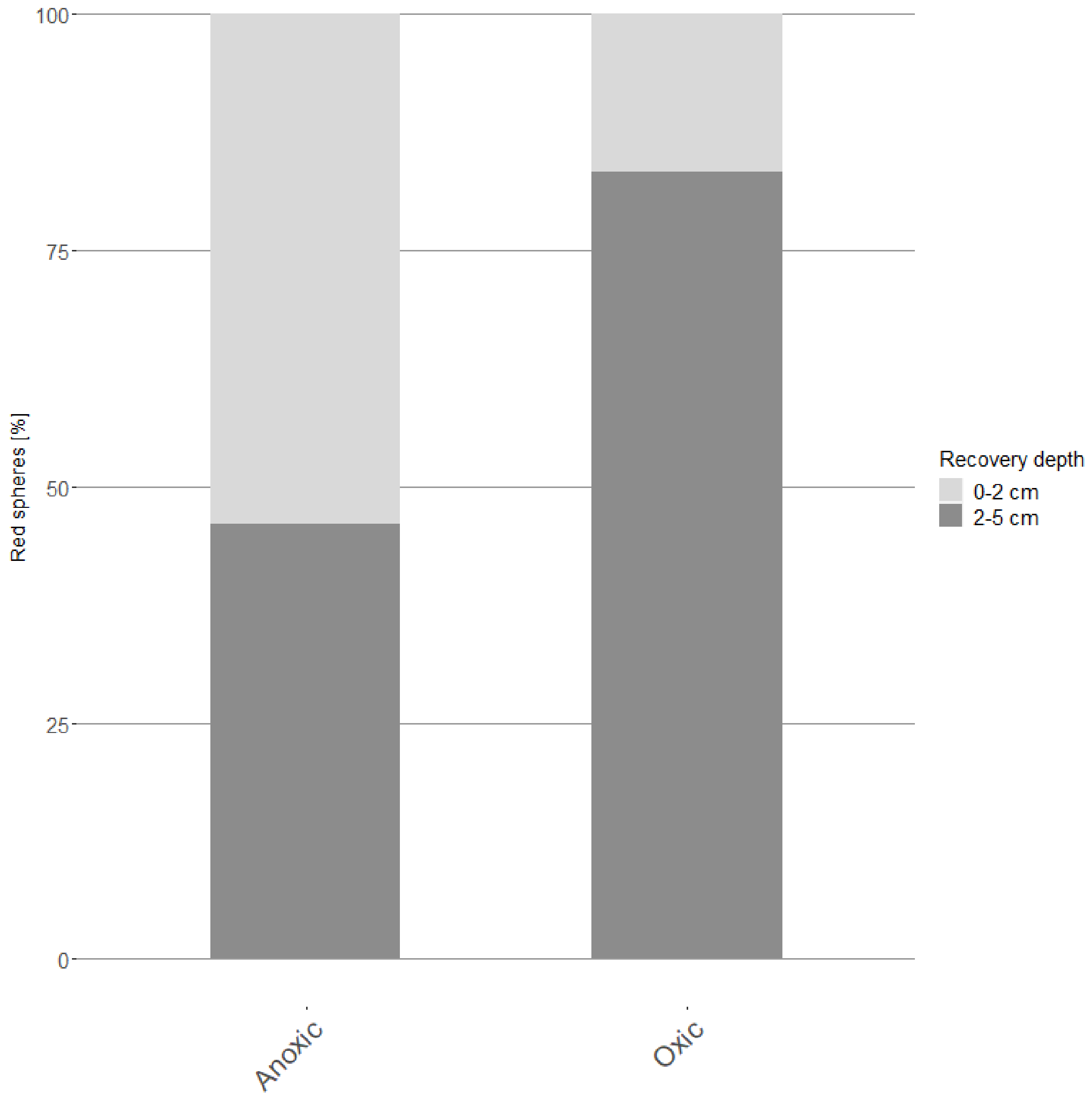

Supplementary Figure 4: Relative depth distribution of the red spheres (300 µM flocs) in the August experiment. Displayed are the means of anoxic (n: 2) and oxic cores (n: 3).

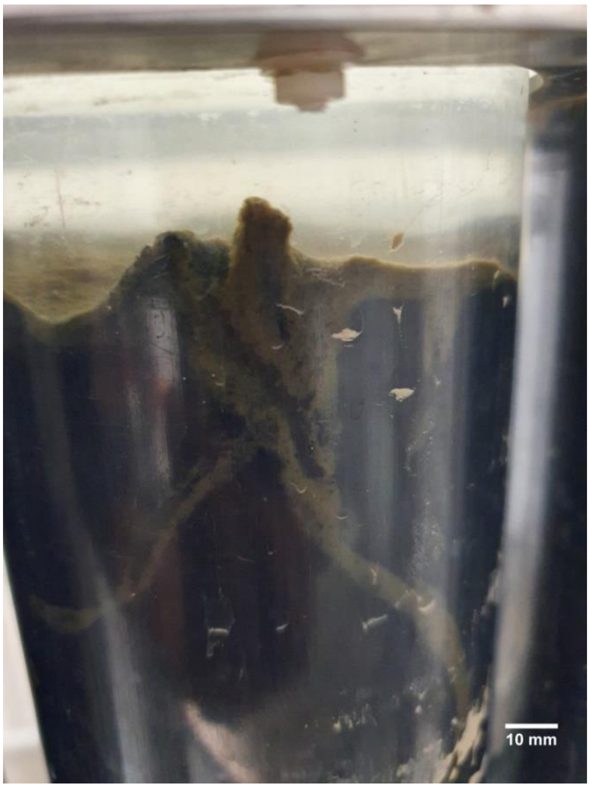

Supplementary Figure 5: Oxic core from the October experiment, showing the burrowing of a chironomid larva close to the walls of the core liner.

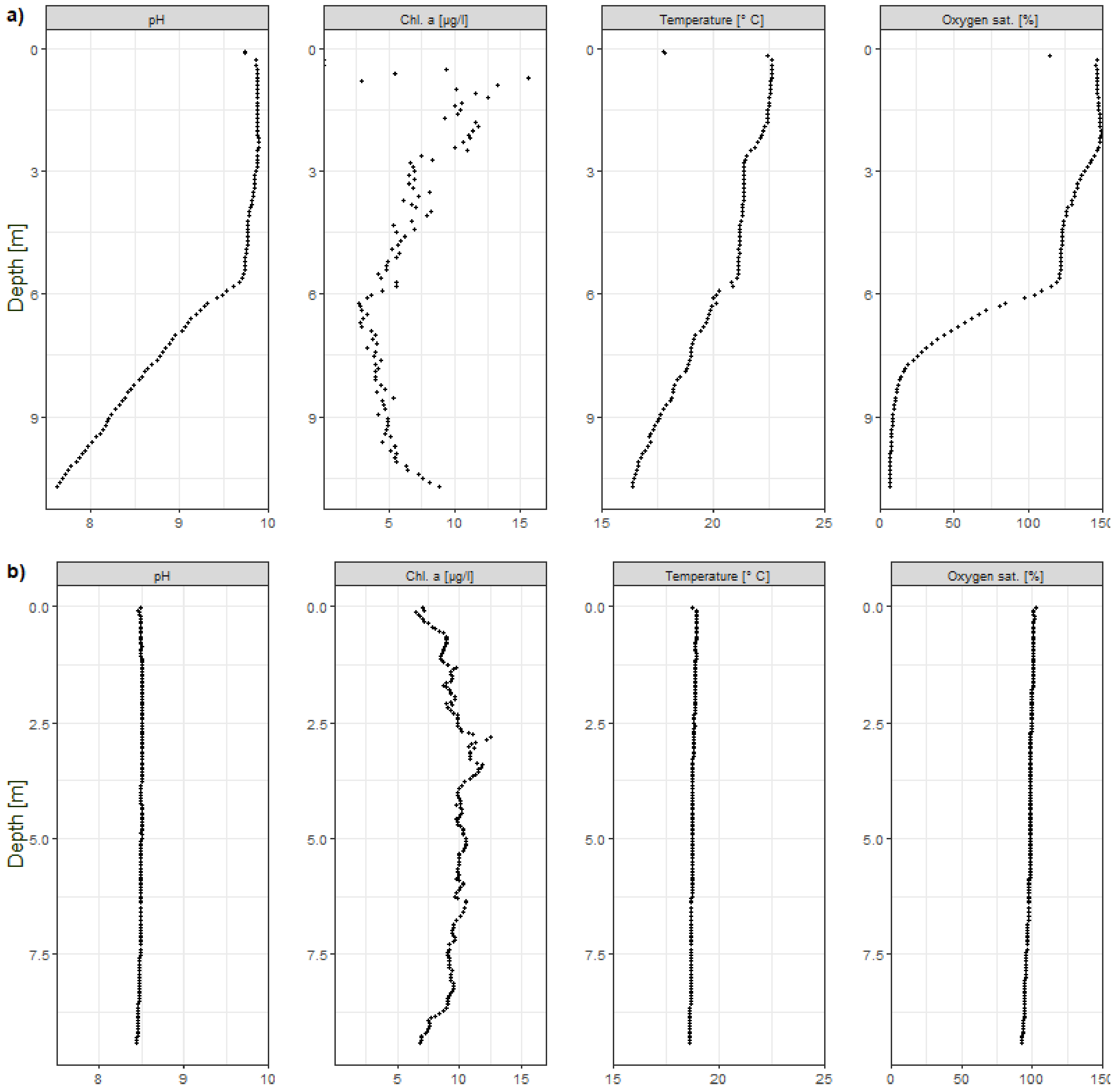

Supplementary Figure 6: Probe profiles showing water parameters in Bautzen reservoir during summer stratification on a) 29th of July 2020 and b) after lake mixing on 19th of October 2020.

Supplementary Table 6: Specifications of the used polyethylene microplastics.

Shape	Size	Color	Density
Sphere	Diameter: 118 ± 6 µm	Red, fluorescent	0.98 g cm^{-3}
Sphere	Diameter: 118 ± 6 µm	Yellow, fluorescent	0.98 g cm^{-3}
Fiber	Length: 4600 µm<br>Diameter: 24 µm	White, transparent	0.92 g cm^{-3}
Fragment<br>10 –100 µm	Equivalent spherical diameter:<br>86 ± 26 µm	White, after staining pink, fluorescent	0.92 g cm^{-3}
Fragment<br>100 – 250 µm	Equivalent spherical diameter:<br>234 ± 55 µm	White, after staining pink, fluorescent	0.92 g cm^{-3}
Fragment<br>250 – 500 µm	Equivalent spherical diameter:<br>466 ± 90 µm	White, after staining pink, fluorescent	0.92 g cm^{-3}
Fragment<br>>500 µm	Equivalent spherical diameter:<br>727 ± 118 µm	White, after staining pink, fluorescent	0.92 g cm^{-3}

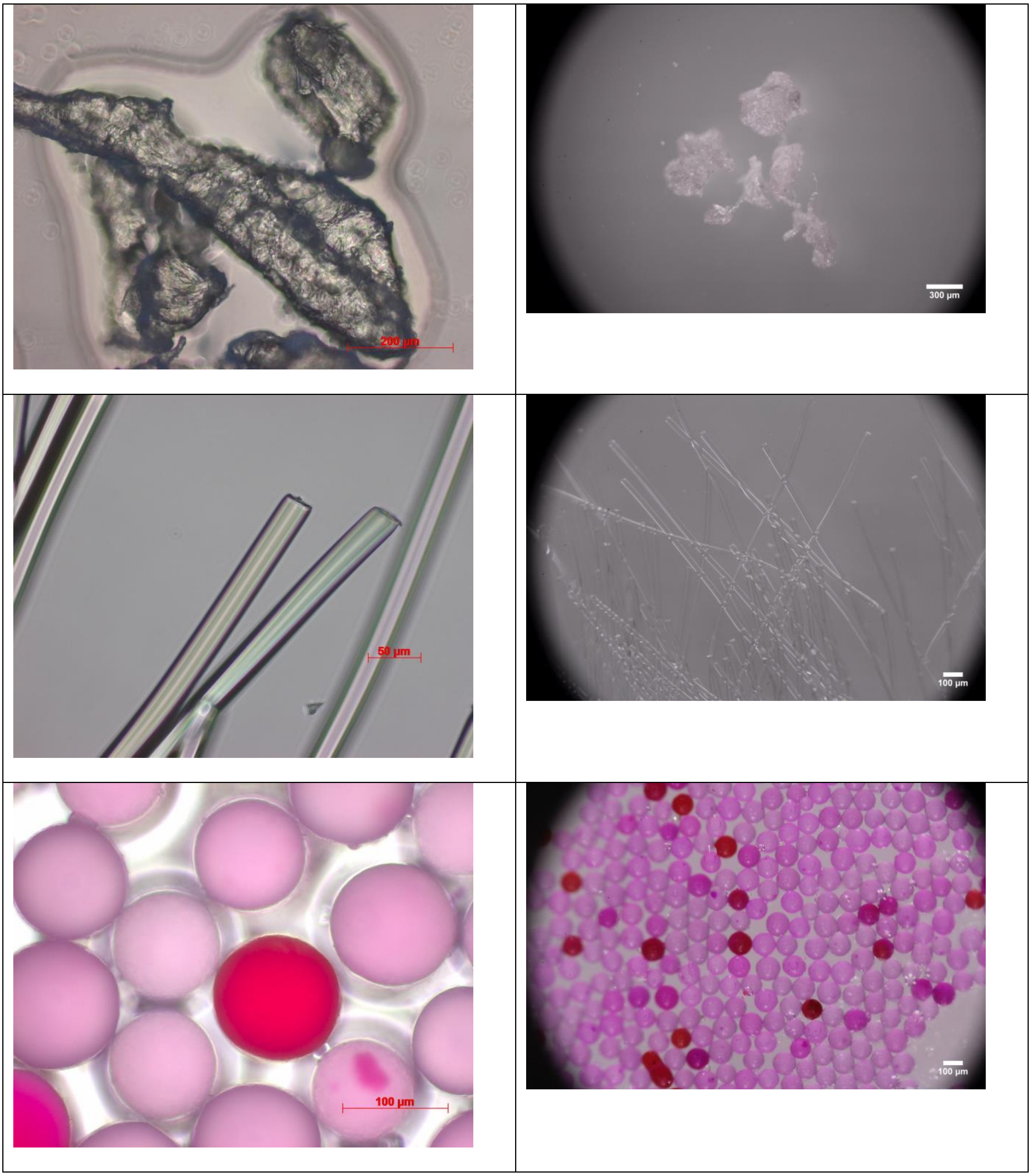

Supplementary Figure 7: Representative images of PE microplastic particles used in the laboratory experiments. From top to bottom, first row: Fragments (magnification left: 200x, right 5x), second row: Fibers (magnification left: 200x, right: 8x), third row: Spheres (magnification left: 200x, right: 8x).

Supplementary Table 7: Excitation and emission wavelengths used for visualization of biofilm components or specific fluorescent molecules via CLSM.

Compound/Component	Excitation	Emission
SybrGreen	561 nm	510-580 nm
Phycobilins (autofluorescence)	633 nm	575-650 nm
Chlorophyll *a* (autofluorescence)	633 nm	650-720 nm
Aleuria Aurantia Lectin with Alexa Fluor 633	621 nm	639 nm
Reflection	490 nm	480-500 nm

References

1. Doeglas, D. J. Grain-Size Indices, Classification and Environment. *Sedimentology* **10**, 83–100 (1968)
